# The Therapeutic Strategies of Regulatory T Cells in Malignancies and Stem Cell Transplantations

**DOI:** 10.1155/2019/5981054

**Published:** 2019-01-01

**Authors:** Rana G. Zaini, Amani A. Al-Rehaili

**Affiliations:** ^1^Deanship of Community Service and Sustainable Development, Taif University, Taif, Saudi Arabia; ^2^College of Applied Medical Sciences, Taif University, Taif, Saudi Arabia

## Abstract

Regulatory T cells (Treg cells) are considered one of the main dynamic cell types within the immune system. Because Treg cells suppress immune responses, they have potential roles in immunological self-tolerance and may help to maintain immune homeostasis. Promoting Treg cell function and increasing their numbers might be useful in treating autoimmune disorders, as well as preventing allograft rejection. However, studies of mice and humans demonstrate that Treg cells promote cancer progression and suppress antitumor immunity. Therefore, suppressing Treg cell function or reducing their numbers could support the immune system's response to pathogenic microorganisms and tumors. As a result, there is great interest in investigating the Treg cells role in the treatment of hematological and nonhematological malignancies. Consequently, Treg cells could be a fundamentally important target for pathologies of the immune system. Targeting effector Treg cells could help to distinguish and selectively decrease these cells while preserving other Treg cells needed to suppress autoimmunity. Currently, a promising way to treat malignancies and other autoimmune disorders is stem cell transplantation. Stem cell transplants (SCT) can help to manage the production of Treg cells and also may produce more efficient Treg cells, thereby suppressing clinical disease progression. Specifically, mature T cells within the engrafted stem cells mediate this SCT beneficial effect. During SCT, the recipient's immune system is replaced with a donor, which allows for improved immune system function. In addition, SCT can protect from disease relapse, as graft-versus-host disease (GvHD) in transplant patients can be protective against cancer recurrence. The current review will define the role of regulatory T cells in treatment of malignancy. Additionally, it will summarize current promising research regarding the utility of regulatory T cells in stem cell transplantation.

## 1. Introduction

The immune system has vital mechanisms that eliminate microbes and diseased cells. At the same time, different mechanisms maintain control of effector cells after their activation by a physiologic inflammatory process [1]. Inflammation must be efficiently regulated to prevent excessive immune reaction. Through cytokine stimulation, CD4+ naïve T cells differentiate into two distinct lineages that have different developmental pathways and unique biological functions. These two types of T cells are helper/effector (Th) and regulatory T (Treg) cells [2, 3]. Effector/helper T cells are the fundamental participants in directing immune reactions. They are crucial in battling pathogens and maintaining immune homeostasis [4, 5]. Moreover, they stimulate further effector immune cells such as CD8^+^ cytotoxic T cells, B cells, and macrophages in order to regulate adaptive immune responses to microorganisms and cancer [6]. Regulatory T (Treg) cells are also identified as suppressor T cells that can suppress possibly harmful Th cells' actions [6]. Gershon first described this in the 1970s [7]. Treg cells are critical in preserving immunological tolerance. They play an essential role in reducing T cell-mediated immunity in order to end the immune effects and to reduce autoreactive T cells [8, 9]. The major differences between Th cells and Treg cells is that effector T cell sets generally promote an immune response through their ability to initiate with immune-enhancing cytokines and then shift to inhibitory cytokines later in their life cycle, whereas Treg cells typically help to moderate and neutralize the immune response (i.e., immune-suppressive) [10]. The greatest noticeable role of Treg cells is maintaining self-tolerance immunity and immune homeostasis by reducing the immune response [7, 11–14]. Thus, any failure in Treg cell function could result an excess of inflammatory and autoimmune diseases [15].

Treg cells are subgroup a group of CD4 T cell compartments that can be originated from the thymus (i.e., called naturally occurring Treg (nTreg) cells) or can be produced from immature T cells in the presence of IL-2 and Transforming growth factor-*β* (TGF-*β*) following the prompt of T cell receptors (i.e., called induced Treg (iTreg) cells) [16]. They are characterized by coexpression of CD4+ and CD25+. These markers are believed to be important in the stimulation of immunological tolerance. Moreover, several surface markers have been reported for the suppression of Treg cells function. These include CD25+, a subunit of IL-2 receptor (IL-2R), CD4+, CTLA-4, CD73+, and CD39+ [17, 18]. The identification and isolation of Treg cells commonly depend on exploiting the CD4+ and CD25+ expression, typically with flow cytometric analysis [19]. Almost a decade ago, the transcription factor Forkhead box P3 (FoxP3) has been detected on Treg cells. FoxP3 has a fundamental role in controlling the process of inflammation [1]. However, CD25+ is not a specific marker of Treg cells because simply expressing CD25+ does not guarantee induction of the suppressor phenotype as they also presented on normal activated T cells [20]. Consequently, it is difficult to distinguish between these cells by flow cytometry only. Nevertheless, FoxP3+ cells have been recently shown that they express a subunit of IL-7R, called CD127, in a notably low density [21]. Unfortunately, the intracellular location of FoxP3 makes it difficult to identify by flow cytometry [22, 23]. Thus, this marker should be identified after cell permeabilization, but this is not practical for routine clinical laboratory testing [22, 23].

However, a study by Baron et al. (2007) suggests that demethylation of the Treg-Specific Demethylated Region stabilizes (TSDR) FOXP3, is a unique phenotype for Treg cells, and does not appear in rapid expression of FOXP3 on activated T cells. Thus, evaluation of these cells by methylation methods provides a good benefit compared to the investigation of protein synthesis and gene expression [24]. Particularly, Baron et al. (2007) used a genome‐wide differential methylation hybridization analysis. Essentially, their study revealed that despite expression of FOXP3 on activated T cells and those treated with Transforming growth factor beta (TGF‐*β*), these cells exposed no demethylation for* FOXP3* DNA, whereas subgroups of Treg that were stable even upon extended* in vitro* expansion remained demethylated. Collectively, they concluded that DNA demethylation constitutes the best current consistent measurement for Treg cells [24].

Detection and quantification of Treg cells within peripheral blood or tissues associated with diseases are considered fundamental processes in understanding the role of these cells in tissue sites. Wieczorek et al. (2009) extended Baron et al. (2007) study and investigated the possibility of using the aforementioned method to measure Treg cells, which looked highly suitable to provide the assay of Treg quantitation [25]. They found that within IL2–treated melanoma patients and patients with various solid tumor such as lung and colon carcinomas, the numbers of Treg cells significantly increased [25]. At the same time, they revealed that application of therapeutic antibodies as immunosuppressive therapy resulted in a substantial decline in Treg from the peripheral blood of transplantation patients [25].

Although Treg cells have an essential role in maintaining immune homeostasis, they also promote cancer progression and suppress antitumor immunity in studies of mice [22, 23, 26, 27] and humans [28–30]. Enhancing the function of Treg cells or increasing their numbers could be valuable in treating allergic and autoimmune disorders, as well as in preventing allograft rejection. On the other hand, suppressing Treg cell function or reducing their numbers could help support the immune system's response against pathogenic microorganisms and tumors [31]. Thus, Treg cells could be fundamentally important in immunopathogenesis, since they may have a role as a therapy for immunological disorders and malignancies.

The current review will debate the regulatory T cells role in treatment of malignancies. It will also summarize the current possible uses of these cells in stem cell transplantation.

## 2. Treg Cells in Animal and Human Studies

It has been previously mentioned in this review that Treg cells have a vital role in the prevention of autoimmunity through their capability to inhibit T cells proliferation and the cytokines production. However, depletion of Treg cells due to a mutation of the gene for transcription factor FoxP3 can lead to serious autoimmune disorders [32, 33]. A study in scurfy mice by Bennett et al. (2001) determined that the loss of FoxP3 protein and nTreg cells due to mutations in the FoxP3 gene led to CD4+ T cells hyperactivation, causing early onset of organ-specific autoimmune pathology [32]. Similarly, the expression of low amounts of FoxP3 protein in Treg cells has been found to be significantly associated with impaired suppressor cell function [34]. Interestingly, mice that overexpress FoxP3 have amplified nTreg cell development, which stops the progression of lymphoproliferative syndrome and type one diabetes in mice deficient in nonobese diabetic (NOD) and cytotoxic T-lymphocyte–associated antigen 4 (CTLA-4) mice, respectively [35].

The relationship of Treg cells and cancer situation is different from autoimmune disease. Enhanced number of Treg cells might support the tumor progression and impact on the disease course; therefore both the proportion and function of Treg cells are essential factors at cancer environment [31, 36]. A study by Viehl et al. (2006) has shown that there is an increase in the Tregs frequency within tumor-bearing mice and the suppression or depletion of these cells can improve their antitumor immunity [37]. A study using a murine fibrosarcoma model showed that elevated Treg cells levels were detected at late stages of cancer, implying they may have a role in cancer progression [35].

Human research has also shown that a genetic defect of the FoxP3 gene can prevent the Treg cells development, leading to ranges of autoimmune diseases and severe allergies [1, 38]. There is accumulative evidence demonstrating that FoxP3+CD25+CD4+Treg cells prevent immune responses to cancerous cells [39] and further demonstrating the Treg cells role in promoting tumour growth through inhibiting vaccine-stimulated antitumor immune reactions and preventing successful tumor control. Thus, the elevated CD4+ CD25^+^ T cells percentage was detected in patients diagnosed with melanoma [40, 41], gastric [42, 43], and ovarian cancers [13, 44, 45]. Similarly, Liyanage et al. (2002) found that the levels of Treg cells were notably greater in patients with breast cancer [46] and pancreatic cancer [46–49].

## 3. Therapeutic Uses of Regulatory T Cells in Cancer

This review will discuss several studies, which investigate the modification of Treg cells as a therapy for malignancy. The most recent studies suggest targeting molecules specific to Treg cells and attempting to either deplete or modify the function of these cells once identified. These targeted molecules include OX-40, CTLA-4, GITR, CCR4, PD-1, LAG3, CD25, and CD15s.

### 3.1. Targeting T Cell Receptor Signalling Molecules and Depleting the Regulatory T Cell Population

#### 3.1.1. Studies of Folate Receptor 4 (FR4)

A possible way to enhance tumor immunity by modifying Treg cells is to target the T cells receptor (TCR) signalling molecules. An example of a TCR signalling molecule is the Folate Receptor 4 (FR4) [50]. FoxP3+Treg cells in rodents express a higher FR4 level as compared to naïve T cells. Additionally, during the process of TCR stimulation, greater proportions of FR4 are upregulated than naïve T cells (i.e., FoxP3−T cells). This enables the activated effector T cells to be easily differentiated from activated Treg cells. Accordingly, an anti-FR4 depleting monoclonal antibody could be valuable to promote immunity of tumors through diminishing activated Treg cells whereas maintaining tumor-reactive effector T cells [51].

### 3.2. Targeting T Cell Signalling Molecules and Modifying Function of the Treg Cell Population

#### 3.2.1. Studies of Glucocorticoid-Induced Tumor Necrosis Factor Receptor (GITR) Surface Molecule

GITR is a molecule highly presented by Treg cells that could be targeted to modulate Treg cell function. Mice studies have demonstrated that application of anti-GITR antibody (nondepleting) can decrease the inhibition activities of Treg cells and increase the effector function of other T cell types to break the self-tolerance immunity [52, 53]. Additionally, this antibody can also trigger antitumor immunity by increasing the amount of IFN-*γ*- yielding CD4+ and CD8+ T cells [54]. Currently, clinical trials have been examined the use of anti-GITR antibodies in patients with progression of solid tumors, such as melanoma [50].

#### 3.2.2. Studies of OX40 Surface Molecule

OX40 (CD134) is a type of the tumor necrosis factor receptor groups. OX40 is present on activated T cells, Treg cells, other lymphoid cells, and nonlymphoid cells [55]. Triggering of OX40 signalling with anti-OX40 mAbs (i.e., agonistic antibody) has been found to reduce the inhibitory activity of Treg cells [55]. Another study showed that administration of anti-OX40 monoclonal Ab promoted robust suppression of tumor progression [56]. The same study showed that OX40 signalling could amend the actions of effector T and Treg cells by reducing the suppressive activity of Treg cells and stimulating the function of effector T cells [56]. Collectively, stimulating OX40 signalling could be useful in regulating the inhibitory effects of Treg cells, thereby preventing tumor growth.

#### 3.2.3. Studies of Combination of Tumor Site-Located CTL-Associated Antigen-4 (CTLA-4)

CTLA-4 is presented on activated T cells as a negative immunomodulator. During immune responses, CTLA-4 provides inhibitory signalling mechanism. It is also particularly expressed by Treg cells and is mediated following the stimulation process of TCR. Although the significance of CTLA-4 for Treg cells role is yet under debate, it has been stated that obstruction of CTLA-4 signalling terminates the Treg cells' suppressive activity [57]. Recently, it has been shown that using conditional knockout mice has demonstrated that lack of CTLA-4 in Treg cells prevents immune system self-tolerance and impairs the inhibitory role of Treg cells in tumor immunity [58, 59]. Furthermore, in mice, selectively blocking CTLA-4 signalling mechanism in non-Treg T cells or Treg cells reveals that CTLA-4 is needed for both Treg cells and activated effector T cells. This blocking enhances tumor suppression by diminishing the suppressor activity of Treg cell and amplifying the function of effector T cell [58, 59]. Mainly, CTLA4 stabilizes the function of CD28 (i.e., the T cell costimulatory receptor). CD28 does not affect the stimulation of T cell unless the TCR is initially engaged by related antigen. After antigen recognition happens, CD28 signalling powerfully increases TCR signalling to activate T cells. CD28 and CTLA4 allocate same ligands: CD80 and CD86. Whereas the particular CTLA4 mechanisms are still not fully understood, because CTLA4 has a much superior overall affinity for both ligands, it has been suggested that its expression on the T cells surface reduces the T cells activation by outweighing CD28 in binding CD80 and CD86 and actively producing suppressive signals to the T cell [59]. The therapeutic use of anti-CTLA4 mAb helps to regulate Treg cells and it possibly serves as promising approach to develop antitumor response. For example, the combined use of anti-GITR mAb and anti-CTLA-4 mAb provoked a high effective response of antitumor than mAb alone, causing regression of advanced stage tumors [54].

## 4. Stem Cell Transplantation as a Therapeutic Approach to Modify Regulatory T Cells in Malignancies

It has been well established that hematopoietic stem cell transplantation (SCT) is useful in hematological malignancies as well as nonmalignant hematological disorders. Chemotherapy and/or radiotherapy destroys a patient's hematopoietic system and enhances immunosuppression to engraft donor stem cells [60]. Donor immune cells facilitate the engraftment of stem cells, protect against infections, and most importantly destroy the remaining hematopoietic cells of the host. In addition, this process protects from disease retrogression during transplantation occurring in patients with leukemia or lymphoma, known as the graft-versus-leukemia effect [60].

The success of SCT depends on the substitution of the recipient's immune system with the immune system cells of donor. Specifically, mature T cells within the engrafted stem cells mediate this SCT beneficial effect [60]. However, the donor's T cells can also attack the recipient's tissues and generate a life-threatening syndrome called graft-versus-host disease (GvHD) [61]. The SCT challenge is to make a balance between the harmful effects and the beneficial T cells, which is currently only insufficiently achieved with immunosuppressive drugs. These Treg cells decrease GvHD whereas maintaining the graft-versus-leukemia (GVL) effect in various mouse model systems. Their use in the clinical trials of SCT may be shortly studied, as their characterization in humans is rapidly progressing [60].

Treg cells have been shown to have potential in preventing GvHD in hematopoietic stem cell transplantation (HSCT). After HSCT, donor T cells that protect patients from infection can also attack the host tissues causing GvHD [61]. It has been postulated that in patients receiving bone marrow infusions, there is a 30% to 60% chance of transferred immune cells producing an immune response against (i.e., GvHD) the recipient's system [62]. A study by Rezvani and Barrett (2008) reported that patients with acute leukemia treated with HSCT showed reconstitution of the immune system after high irradiation doses [61]. Thus, there is great interest in preventing GvHD without affecting the donor T cells' ability to protect from pathogens [63, 64]. Studies by Di Ianni et al. (2011) showed that 26 patients out of 28 had successful stem cell engraftment with only two of the patients developing greater than or equal to grade two GvHD [63]. Similarly, Martelli et al. (2014) found that donor engraftment was 95% successful in the 43 patients who received Treg cells four days prior to HSCT. In addition, this study showed that only 15% of patients developed grade two GvHD [64]. Together, more researches are needed in terms of potential use of stem cell transplantation in curing a variety of malignancies and other autoimmune disorders.

## 5. Conclusion

There are accumulating data showing that CD25+CD4+Treg cells antagonistically suppress antitumor immune effects in different types of malignancies. These cells might be chemoattracted to tumor-associated macrophages (TMEs) and appear in high levels in tumors. Currently, clinical studies are investigating depletion and modification of the CD25+CD4+Treg cells by different methods. These therapies include anti-GITR, anti-OX40, and anti-CTLA-4 antibodies. Synergistic antitumor effects can be achieved by using combination treatment to target non-Treg and Treg cells, which changes the balance between the two cell populations. This combination therapy can suppress Treg cells and simultaneously increase effector T cell activity. Accordingly, the new cancer treatments with regard to Treg cells management could include blocking their trafficking into tumors, depletion, or diminishing their differentiation and mediating their mechanisms. More work is still needed to create proper protocols, including correct biomarkers, for monitoring of treatment efficacy. Currently, stem cell transplantation promises to treat a variety of malignancies and other autoimmune disorders. To suppress disease progression, with early management of production and efficacy of Treg cells, these cells are actively being investigated as a way to improve SCT. Collectively, it is now clear that there is proof from both animal and human studies that the Treg cells have a pivotal role at cancer context. They have a substantial role in cancer progression, and they have a significant role destroying tumor immunity. Thus, the future researches in terms of malignancies treatments should focus on developing new clinical approaches to decrease their regulatory effects, along with the essential goal of enhancing their antitumor immunity.
